# The regulation of lipid and carbohydrate storage by the splicing factor gene *snRNP-U1-70K* in the *Drosophila* fat body

**DOI:** 10.17912/micropub.biology.000580

**Published:** 2022-05-30

**Authors:** Hamza Shabar, Justin R. DiAngelo

**Affiliations:** 1 Division of Science, Penn State Berks, Reading, PA, USA

## Abstract

Excess triglycerides from the diet are stored in structures called lipid droplets in adipose tissue. Genome-wide RNAi screens have identified mRNA splicing factors as important for lipid droplet formation; however, the full complement of splicing factors that regulate lipid storage is not known. Here, we characterize the role of *snRNP-U1-70K* , the gene encoding for a splicing protein involved in recognizing the 5’ splice site in introns, in regulating lipid and carbohydrate storage in the *Drosophila* fat body. Decreasing *snRNP-U1-70K* specifically in the fly fat body resulted in less triglyceride, glycogen, and glucose in each fat body cell. Consistent with these decreased nutrient storage phenotypes, *snRNP-U1-70K-RNAi* flies ate less, providing a potential cause for less lipid and carbohydrate storage in these flies. These data further support the role of mRNA processing in regulating metabolic homeostasis in *Drosophila* .

**Figure 1. Decreasing snRNP-U1-70K in the fat body results in reduced carbohydrate and lipid storage.**
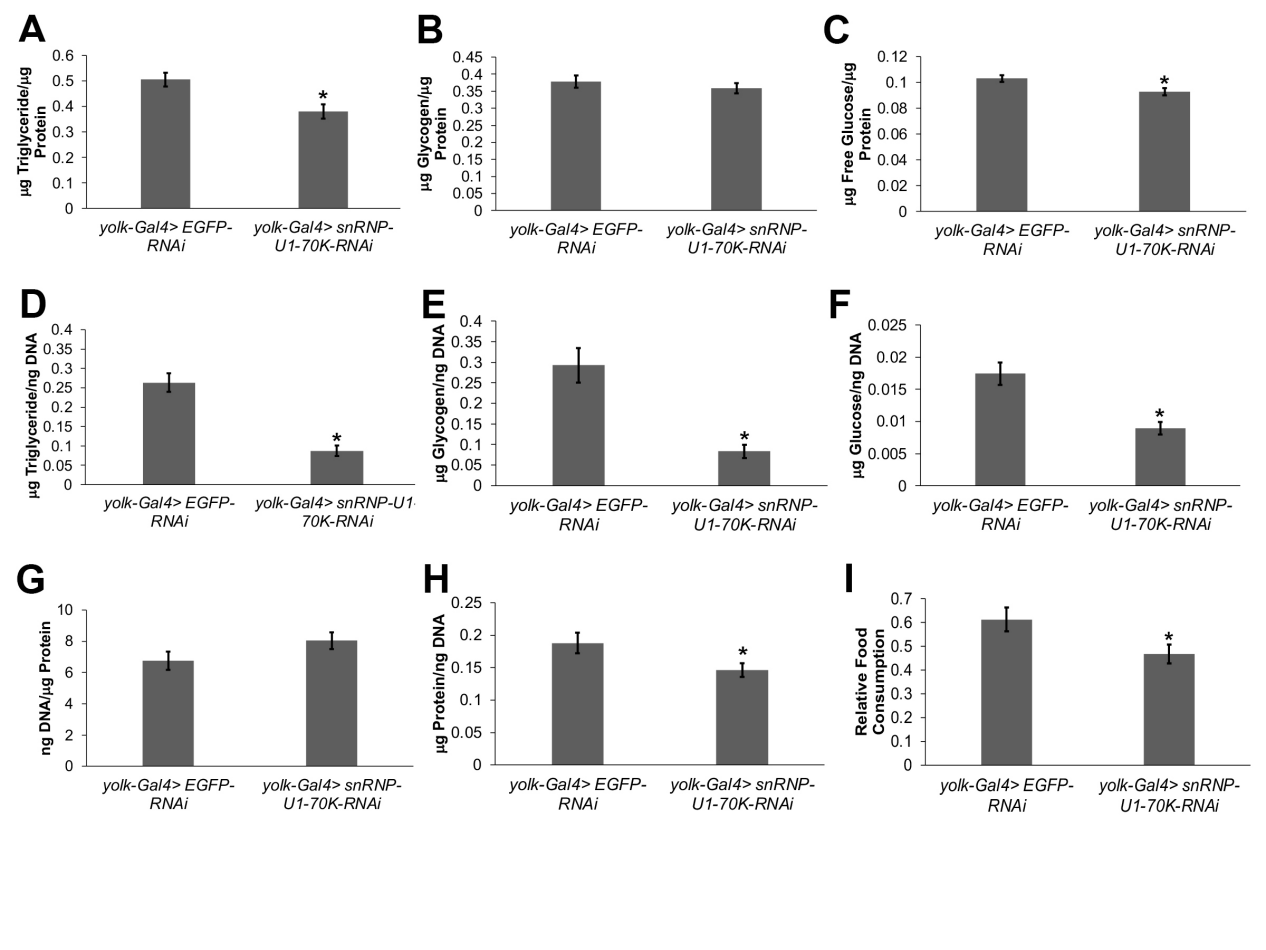
**(A)** Triglyceride, **(B)** glycogen, and **(C)** free glucose, all normalized by total protein content, were measured in one-week old mated female *yolk-Gal4>snRNP-U1-70K-RNAi* flies (n=48) and compared to the *yolk-Gal4>EGFP-RNAi* controls (n=38). **(D)** Triglyceride, **(E)** glycogen, and **(F)** glucose, all normalized by fat body DNA content, **(G)** fat body DNA content normalized by total protein content, and **(H)** fat body protein content normalized by DNA content were measured in cuticles with fat bodies attached dissected from one-week old mated female *yolk-Gal4>snRNP-U1-70K-RNAi* flies (n=35) and compared to the *yolk Gal4>EGFP-RNAi* controls (n=34). **(I)** Food consumption over a 24-hour period was measured in one-week old mated female *yolk-Gal4>snRNP-U1-70K-RNAi* flies (n=38) and compared to female *yolk-Gal4>EGFP-RNAi* flies (n=43), using the CAFÉ assay. Each experiment was performed 3-4 times and error bars represent standard error of the mean of the data generated from all samples from all experiments. *p<0.05 as determined by a t-test.

## Description

Evolutionarily, animals consume food to make energy and the excess calories are stored as fats and carbohydrates. This drive to store excess nutrients proves to be useful in a population that may face possible threats of famine or a scarcity of resources. Today, where an abundance of nutrient-rich food is readily available, the high consumption of these foods can be the underlying cause for metabolic diseases such as obesity. A better understanding of the mechanisms that result in excess fat storage can be crucial for better treating obesity-related diseases.

The fruit fly, *Drosophila melanogaster* , is a commonly used model organism in biological fields due to their short lifespan, well annotated genome, and high conservation to humans. In addition, the fly has recently emerged as a good model for studying metabolic processes as flies have a tissue highly analogous to that of human adipose tissue called the fat body (Heier et al. 2021; Musselman and Kühnlein 2018). To identify the genes that are important for regulating lipid metabolism in *Drosophila* , genome-wide RNA interference (RNAi) screens were performed to measure lipid droplet size and number in cultured cells (Beller et al. 2008; Guo et al. 2008). Decreasing the expression of genes that play a role in RNA processing resulted in fewer lipid droplets. However, whether these genes regulate lipid metabolism *in vivo* is still unknown.

After transcription, noncoding intron sequences are removed from the RNA transcript by proteins called small nuclear ribonucleoproteins (snRNPs). SR (serine/arginine rich) proteins, splicing factors that contain a domain rich in serines and arginines, are involved with pre-mRNA splicing by helping to identify and recruit snRNPs to specific splice sites. Therefore, SR proteins are important regulators of gene expression. The SR protein snRNP-U1-70K was identified to play an integral role in RNA splicing by helping to target the U1 snRNP to the 5’ splice site in humans (Cao and Garcia-Blanco 1998; Jamison et al. 1995; Kohtz et al. 1994). However, whether snRNP-U1-70K has any metabolic functions is not known. Previous work from our laboratory has shown that decreased expression of *snRNP-U1-70K* in the *Drosophila* fat body results in decreased triglyceride storage (Gingras et al. 2014). However, the mechanisms whereby *snRNP-U1-70K* regulates triglyceride storage is still unclear.

In this study, *snRNP-U1-70K* was decreased specifically in the fat body using RNAi and the resulting metabolic phenotypes such as carbohydrate and lipid storage and feeding were characterized. *snRNP-U1-70K* RNA levels were decreased by approximately 40% in *snRNP-U1-70K-RNAi* fat bodies compared to controls as measured by quantitative PCR (p=0.05 as determined by t-test, n=4). Triglycerides, glycogen, and free glucose were measured in whole animals and normalized to total protein content. The triglyceride ( **Fig. 1A** ) and free glucose ( **Fig. 1C** ) to protein ratios in fat body-specific *snRNP-U1-70K-RNAi* flies were decreased compared to the *EGFP-RNAi* controls. However, there was no difference in the glycogen to protein ratio between the *snRNP-U1-70K-RNAi* flies as compared to the controls ( **Fig. 1B** ). The decreased storage of triglycerides and free glucose in *snRNP-U1-70K-RNAi* flies could be a result of fewer fat cells being produced, less fat and/or glucose being stored per fat cell, or a combination of both. To differentiate among these possibilities, fat bodies were dissected from *snRNP-U1-70K-RNAi* and control flies and total fat body DNA content was measured and used as a surrogate measurement of cell number as previously described (DiAngelo and Birnbaum 2009). In addition, triglyceride, glycogen and free glucose levels were measured in these fat bodies and normalized by total DNA content to determine the amount of each of these macromolecules stored per cell. The DNA normalized triglyceride ( **Fig. 1D** ), glycogen ( **Fig. 1E)** , and glucose ( **Fig. 1F)** were all decreased in the dissected fat body-specific *snRNP-U1-70K-RNAi* flies compared to the controls. The fat body DNA content normalized to protein content in *snRNP-U1-70K-RNAi* flies was similar compared to the controls ( **Fig. 1G** ); however, protein normalized to DNA, a measure of cell size (Marguerat and Bähler 2012), in *snRNP-U1-70K-RNAi* flies was less than controls ( **Fig. 1H** ). Together, these data suggest that decreasing *snRNP-U1-70K* in the fat body results in smaller cells being present in the fat body, and less nutrients being stored in each of those cells.

It is also possible that the decrease in the nutrients stored in each fat body cell could be due to lower consumption of food in *snRNP-U1-70K-RNAi* flies. To test this hypothesis, feeding was monitored over a 24-hour period using the CAFÉ assay. Consistent with this hypothesis, *snRNP-U1-70K-RNAi* flies ate less than the controls ( **Fig. 1I** ), suggesting that decreased food consumption results in less carbohydrate and lipid storage in fat body cells.

In agreement with previous work (Gingras et al. 2014), this study has shown that fat body-specific knockdown of *snRNP-U1-70K* decreased triglyceride storage and free glucose in *Drosophila* . In addition, the fat body protein/DNA, triglyceride/DNA, glycogen/DNA, and free glucose/DNA ratios were all decreased in *snRNP-U1-70K-RNAi* flies suggesting that *snRNP-U1-70K* functions in the fat body to regulate fat cell size and the amount of carbohydrates and lipids that are being stored in each of those cells. Interestingly, previous studies from our laboratory have shown that reducing the levels of other SR proteins ( *9G8* , *Tra2* , *SF2* and *RBP-1* ) results in a triglyceride accumulation phenotype, which is the opposite of what occurs in *snRNP-U1-70K-RNAi* flies (Bennick et al. 2019; Gingras et al. 2014; Mikoluk et al. 2018). This suggests that *snRNP-U1-70K* may be targeting a different subset of metabolic genes to regulate fat body nutrient storage than *9G8* , *Tra2* , *SF2* and *RBP-1* and further experimentation is needed to address this possibility. Together, the data presented in this study has provided further support for the link between splicing factors and the regulation of lipid metabolism.

## Methods

*Fly husbandry.* Flies were grown at 25°C in an incubator on a 12h:12h light:dark cycle. Flies were fed a standard sugar-yeast-cornmeal fly food (9 g *Drosophila* agar (Genesee Scientific), 100 mL Karo Lite Corn Syrup, 65 g cornmeal, 40 g sucrose, and 25 g whole yeast in 1.25 L water). Homozygous virgin *yolk-Gal4* flies were crossed to either homozygous *UAS-snRNP-U1-70K-RNAi* or *UAS-EGFP-RNAi* male flies and female progeny were mated with male siblings and aged for approximately one week before being used for the experiments described here.

*Triglyceride, Glycogen, Free Glucose, Protein, and DNA Assays.* Protein, triglyceride, glycogen, and free glucose were measured in 2 one-week old whole female flies or cuticles with fat bodies attached dissected from 4 one-week old female flies, whereas DNA was only measured in the dissected fat body samples. Whole flies or dissected fat bodies were homogenized by sonication in lysis buffer (140mM NaCl, 50mM Tris-HCl, 0.1% Triton-X and protease inhibitor cocktail (Roche)). The Pierce BCA Assay kit (Thermofisher) was used according to manufacturer’s instructions to measure protein concentrations. The Infinity Triglyceride reagent (Thermofisher) was used according to manufacturer’s instructions to measure triglyceride concentrations. Pointe Scientific Glucose Oxidase kit (Thermofisher) was used according to manufacturer’s instructions to measure both free glucose and glycogen concentration. Total glucose was measured by adding 8 mg/mL amyloglucosidase (Sigma) diluted in 0.2M citrate buffer, pH 5.0 and incubating at 37°C for 2 hours. Free glucose was subtracted from total glucose to determine glycogen concentration. The DNA High Sensitivity Reagent Kit (Thermofisher) was used according to manufacturer’s instructions to measure DNA concentration.

*RNA Isolation and Quantitative PCR.* 15 cuticles with fat bodies attached dissected from one-week old females flies were homogenized in VWR Life Science RiboZol RNA Extraction Reagent (VWR) and total RNA was isolated according to manufacturer's instructions. 5 μg of total RNA was DNase treated with the Turbo DNA-free kit (Thermofisher) and 0.25 μg of DNase-treated RNA was reverse transcribed using qScript-XLT cDNA Supermix (Quanta Biosciences) according to manufacturer's instructions. Quantitative PCR was performed in 25 μl reactions with 1 μl of cDNA, 1x Perfecta SYBR Supermix (Quanta Biosciences) and 200 nM primers. The cycling conditions for the PCR were as follows: 3 min at 95°C; 40 cycles of 95°C for 30 seconds, 60°C for 1 minute, and 72°C for 30 seconds; followed by a melt curve. *snRNP-U1-70K* levels were normalized by *RpL32* levels. The primer sequences used were: *snRNP-U1-70K* (sense 5'-GGACCCCACAGAGATCAAAA-3' and antisense 5'-CTCGTGCTCGTACTCGATGA-3') and *RpL32* (sense 5'-GACGCTTCAAGGGACAGTATCTG-3' and antisense 5'-AAACGCGGTTCTGCATGAG-3').

*Feeding Assay.* Food consumption was measured using the CAFÉ assay as previously described (Ja et al. 2007). Briefly, 3 female flies were added to vials containing 1% agar (vials without flies were used to control for evaporation). Flies were fed 5% sucrose using 5 μl capillary tubes over a 24-hour period.

## Reagents

**Table:** 

**Strain**	**Genotype**	**Available from**
yolk-Gal4	y[1] w[*]; P{w[+mC]=yolk-GAL4}2	Bloomington Stock Center #58814
UAS-EGFP-RNAi	y[1]sc[*]v[1]; P{y[t7.7]v[t1.8] VALIUM20-EGFP}attP2	Bloomington Stock Center #35782
UAS-snRNP-U1-70K-RNAi	y[1] sc[*] v[1] sev[21]; P{y[+t7.7] v[+t1.8]=TRiP.HMS00274}attP2	Bloomington Stock Center #33396

## References

[R1] Beller M, Sztalryd C, Southall N, Bell M, Jäckle H, Auld DS, Oliver B (2008). COPI complex is a regulator of lipid homeostasis.. PLoS Biol.

[R2] Bennick RA, Nagengast AA, DiAngelo JR (2019). The SR proteins SF2 and RBP1 regulate triglyceride storage in the fat body of Drosophila.. Biochem Biophys Res Commun.

[R3] Cao W, Garcia-Blanco MA (1998). A serine/arginine-rich domain in the human U1 70k protein is necessary and sufficient for ASF/SF2 binding.. J Biol Chem.

[R4] DiAngelo JR, Birnbaum MJ (2009). Regulation of fat cell mass by insulin in Drosophila melanogaster.. Mol Cell Biol.

[R5] Gingras RM, Warren ME, Nagengast AA, Diangelo JR (2013). The control of lipid metabolism by mRNA splicing in Drosophila.. Biochem Biophys Res Commun.

[R6] Guo Y, Walther TC, Rao M, Stuurman N, Goshima G, Terayama K, Wong JS, Vale RD, Walter P, Farese RV (2008). Functional genomic screen reveals genes involved in lipid-droplet formation and utilization.. Nature.

[R7] Heier C, Klishch S, Stilbytska O, Semaniuk U, Lushchak O (2021). The Drosophila model to interrogate triacylglycerol biology.. Biochim Biophys Acta Mol Cell Biol Lipids.

[R8] Ja WW, Carvalho GB, Mak EM, de la Rosa NN, Fang AY, Liong JC, Brummel T, Benzer S. 2007. Prandiology of Drosophila and the CAFE assay. Proc Natl Acad Sci U S A 104: 8253-6. 10.1073/pnas.0702726104 PMC1899109 17494737

[R9] Jamison SF, Pasman Z, Wang J, Will C, Lührmann R, Manley JL, Garcia-Blanco MA (1995). U1 snRNP-ASF/SF2 interaction and 5' splice site recognition: characterization of required elements.. Nucleic Acids Res.

[R10] Kohtz JD, Jamison SF, Will CL, Zuo P, Lührmann R, Garcia-Blanco MA, Manley JL (1994). Protein-protein interactions and 5'-splice-site recognition in mammalian mRNA precursors.. Nature.

[R11] Marguerat S, Bähler J (2012). Coordinating genome expression with cell size.. Trends Genet.

[R12] Mikoluk C, Nagengast AA, DiAngelo JR (2017). The splicing factor transformer2 (tra2) functions in the Drosophila fat body to regulate lipid storage.. Biochem Biophys Res Commun.

[R13] Musselman LP, Kühnlein RP (2018). *Drosophila*
as a model to study obesity and metabolic disease.. J Exp Biol.

